# Supplementary material for: The Spread of Peste Des Petits Ruminants Virus Lineage IV in West Africa
Source: Animals (Basel). 2023 Apr 6;13(7):1268. doi: 10.3390/ani13071268 (PMC10093634; doi:10.3390/ani13071268)
Supplement: Supplementary file 1 [file animals-13-01268-s001.zip › animals-2269101-supplementary.pdf]

Table S1 Description of samples analysed in this study

#	Country	Sample	Host	Location	Sample type	Date	Result	GT*	GenBank
1	Burkina Faso	2113608012	Sheep	Loumbila	nasal swab	21/12/2021	+	IV	OQ448870
2	Burkina Faso	2113608013	Sheep	Loumbila	nasal swab	21/12/2021	+	IV	OQ448871
3	Burkina Faso	BF3	Sheep	Loumbila	nasal swab	21/12/2021	-	-	-
4	Burkina Faso	BF4	Sheep	Loumbila	nasal swab	21/12/2021	-	-	-
5	Burkina Faso	2113608018	Sheep	Loumbila	nasal swab	21/12/2021	+	IV	OQ448872
6	Burkina Faso	BF6	Sheep	Loumbila	nasal swab	21/12/2021	-	-	-
7	Burkina Faso	2113608023	Sheep	Loumbila	nasal swab	21/12/2021	+	IV	OQ448873
8	Burkina Faso	1900990099	Sheep	Sabou	nasal swab	18/02/2019	+	II	OQ448868
9	Burkina Faso	1900990100	Sheep	Sabou	nasal swab	18/02/2019	+	II	OQ448869
10	Burkina Faso	BF10	Sheep	Piela	nasal swab	03/12/2020	-	-	-
11	Côte d'Ivoire	XX1	Goat	Bouaké	organs	15/06/2015	N/D	-	-
12	Côte d'Ivoire	XX2	Goat	Bouaké	organs	15/06/2015	+	IV	OQ215762
13	Côte d'Ivoire	XX3	Goat	Biankouma	swab	03/03/2017	+	IV	OQ215763
14	Côte d'Ivoire	XX4	Goat	Biankouma	swab	03/03/2017	-	-	-
15	Côte d'Ivoire	XX5	Goat	Biankouma	swab	03/03/2017	+	N/D	-
16	Côte d'Ivoire	XX6	Goat	Grand Yapo	swab	17/12/2013	+	II	OQ215764
17	Côte d'Ivoire	XX7	Goat	Grand Yapo	swab	17/12/2013	+	II	OQ215765

18	Côte d'Ivoire	XX8	Sheep	Dimbokro	organs	12/06/2018	+	II	OQ215766
19	Côte d'Ivoire	XX9	Goat	Agboville	wab	04/02/2018	+	IV	OQ215767
20	Côte d'Ivoire	XX10	Goat	Bocanda	organs	06/03/2021	+	IV	OQ215768
21	Côte d'Ivoire	BN1	Goat	Bongouanou	swab	12/2018	+	IV	OQ215769
22	Côte d'Ivoire	BN2	Goat	Bongouanou	swab	12/2018	+	IV	OQ215770
23	Côte d'Ivoire	BN3	Goat	Bongouanou	swab	12/2018	+	IV	OQ215771
24	Côte d'Ivoire	BN4	Goat	Bongouanou	swab	03/2021	+	IV	OQ215772
25	Côte d'Ivoire	BN5	Goat	Bongouanou	swab	04/2021	+	IV	OQ215773
26	Côte d'Ivoire	BN6	Goat	Bongouanou	swab	04/2021	+	IV	OQ215774
27	Côte d'Ivoire	BC1	Goat	Bocanda	swab	04/2021	+	IV	OQ215775
28	Côte d'Ivoire	BC2	Goat	Bocanda	swab	04/2021	+	-	-
29	Côte d'Ivoire	BC3	Goat	Bocanda	swab	04/2021	+	IV	OQ215776
30	Côte d'Ivoire	BC4	Goat	Bocanda	swab	04/2021	+	IV	OQ215777
31	Côte d'Ivoire	BC5	Goat	Bocanda	swab	04/2021	+	IV	OQ215778
32	Ghana	GG22	Goat	Tse Ado	nasal swab	26/08/2022	-	-	-
33	Ghana	NB22	Sheep	Navrongo	nasal swab	10/08/2022	+	IV	OQ281756
34	Ghana	BB22	Goat	Denyira	nasal swab	05/08/2022	-	-	-
35	Guinea	1OVF	Sheep	Boké	swab	03/04/2022	-	-	-
36	Guinea	60OVF	Sheep	Lola	swab	16/12/2021	+	IV	OQ215759
37	Guinea	7CapF	Goat	Samoyé	swab	16/09/2022	+	IV	OQ215761

38	Guinea	5OVM	Sheep	Lola	swab	16/12/2021	+	IV	OQ215758
39	Guinea	9OVF	Sheep	N'Zérékoré	swab	29/09/2022	-	N/A	N/A
40	Guinea	14OVF	Sheep	Dinguiraye	swab	23/02/2022	+	II	OQ215760
41	Guinea	6CapM	Goat	Kankan	swab	26/07/2021	+	IV	OQ215757
42	Guinea	12OVF	Sheep	Dinguiraye	swab	13/01/2022	-	-	-
43	Guinea	4CapM	Goat	Lola	swab	26/04/2021	-	-	-
44	Guinea	5CapF	Goat	Dinguiraye	swab	13/01/2022	-	-	-
