# Supplementary figures and images for: The Spread of Peste Des Petits Ruminants Virus Lineage IV in West Africa
Source: Animals (Basel). 2023 Apr 6;13(7):1268. doi: 10.3390/ani13071268 (PMC10093634; doi:10.3390/ani13071268)

Tree scale: 0.1

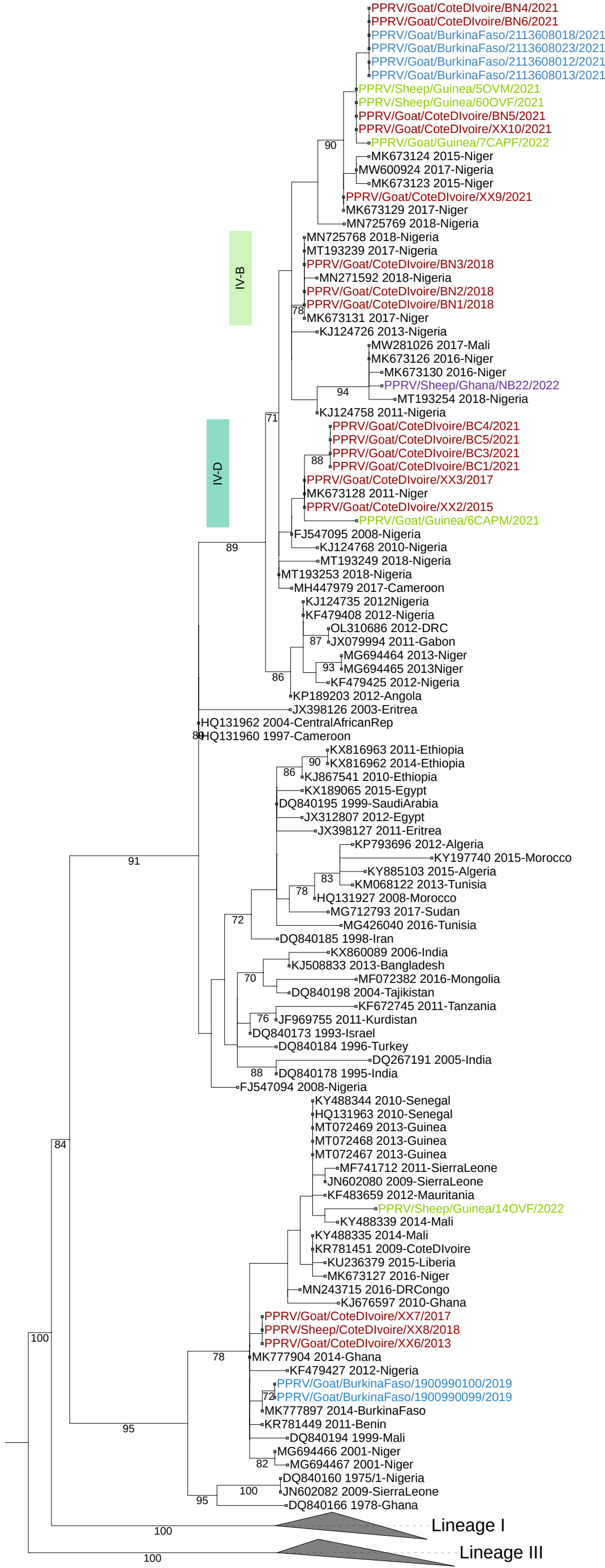

IV-A

IV-C

IV-B

IV-D

Lineage IV

Lineage II

Lineage I

Lineage III

Supplement: Supplementary file 1 [file animals-13-01268-s001.zip › animals-2269101-supplementary Fig S1.pdf]
